# Acquired Leukocyte Inclusion Bodies Resembling Döhle Bodies During Acute Cholangitis

**DOI:** 10.4274/tjh.2017.0121

**Published:** 2017-12-01

**Authors:** Gökhan Özgür, Musa Barış Aykan, Murat Yıldırım, Selim Sayın, Ahmet Uygun, Cengiz Beyan

**Affiliations:** 1 Gülhane Training and Research Hospital, Department of Hematology, Ankara, Turkey; 2 Health Sciences University, Gülhane Faculty of Medicine, Department of Internal Medicine, Ankara, Turkey; 3 Health Sciences University, Gülhane Faculty of Medicine, Department of Gastroenterology, Ankara, Turkey; 4 TOBB University of Economics and Technology Faculty of Medicine, Department of Internal Medicine, Ankara, Turkey

**Keywords:** Cholangitis, Döhle bodies, May-Hegglin anomaly

## To The Editor,

**Figure 1:**
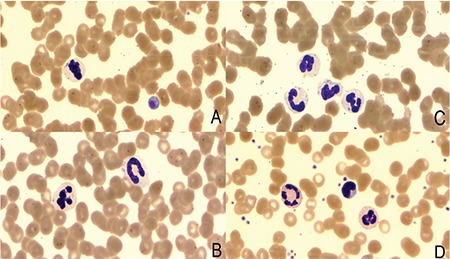
A) Inclusion bodies in neutrophils and macrothrombocyte; B), C) inclusion bodies in neutrophils; D) peripheral blood smear after treatment.

A 66-year-old woman was admitted to the gastroenterology department with epigastric pain, nausea, and subicterus. Her complaints had begun 6 h earlier. Her abdomen was soft and flat, with localized tenderness on palpation in the right subcostal area. Laboratory studies revealed a white cell count of 17.9x10^9^/L, hemoglobin concentration of 14.4 g/dL, and platelet count of 48x10^9^/L, and they were notable for elevated serum cholestatic enzymes. The abdominal ultrasound was remarkable for cholangitis. The patient received broad-spectrum antibiotics. A peripheral blood smear examination, performed to evaluate thrombocytopenia, revealed the presence of blue intracytoplasmic inclusions in neutrophils (Figure 1A-C). On the 11^th^ day of treatment, her blood smear was examined once again and the Döhle body-like inclusions were resolved (Figure 1D).

May-Hegglin anomaly is an uncommon autosomal dominant abnormality characterized by large, basophilic inclusion bodies (resembling Döhle bodies) in neutrophils [1,2]. Döhle bodies can be seen in bacterial infections. Hematologic findings of systemic diseases may be confused with hematological diseases such as May-Hegglin anomaly. We thought that the granules were Döhle bodies due to cholangitis. The disappearance of the inclusion bodies upon treatment is important in differential diagnosis.
